# Reverse Shapiro Syndrome Presenting as Fever of Unknown Origin: A Case Report and Review of the Literature

**DOI:** 10.7759/cureus.90726

**Published:** 2025-08-22

**Authors:** Evangelia Kotsi, Konstantinos Thomas, Myrto Palkopoulou, Maria Papavdi, Pinelopi Kaparou, Alexandros Pelekanos, Melanie Deutsch, Dimitrios Vassilopoulos, Emmanouil Koullias

**Affiliations:** 1 2nd Department of Internal Medicine, National and Kapodistrian University of Athens, Hippokration General Hospital, Athens, GRC; 2 4th Department of Internal Medicine, National and Kapodistrian University of Athens, Attikon General University Hospital, Athens, GRC; 3 Outpatient Neurology Clinic, Hippokration General Hospital, Athens, GRC

**Keywords:** agenesis of corpus callosum, dopaminergic pathway, episodic hypothermia, fever of unknown origin, hyperthermia, hypothalamus, reverse shapiro syndrome, shapiro syndrome, thermoregulation

## Abstract

Reverse Shapiro syndrome (RSS) is an exceptionally rare neurological disorder characterized by recurrent episodes of hyperthermia in the context of agenesis of the corpus callosum (ACC), in contrast to the hypothermic episodes seen in classic Shapiro syndrome (SS). The exact pathophysiology remains unclear; however, hypothalamic dysregulation, neurotransmitter imbalances, and melatonergic involvement are believed to play key roles. Herein, we present a case of RSS in a 33-year-old male patient with a medical history of partial agenesis of the corpus callosum and spastic tetraparesis with persistent episodes of unexplained high fever leading to multiple hospitalizations. Endocrine and infectious causes were excluded. Both antibiotics and supportive management failed to resolve the symptoms, whereas levodopa administration led to complete remission of symptoms. Fewer than 10 cases have been reported in the literature to date. Management in reported cases was largely supportive, with mixed responses to pharmacological agents, such as dopamine agonists and serotonin antagonists. Given the potential for diagnostic delay and its impact on patients' quality of life, awareness of RSS is essential in cases of fever of unknown origin, particularly in patients with known or suspected congenital brain anomalies.

## Introduction

Reverse Shapiro syndrome (RSS) is a rare neurological disorder characterized by agenesis of the corpus callosum (ACC) and thermoregulatory dysfunction, resulting from damage to the anterior preoptic nuclei of the hypothalamus, leading to episodes of hyperthermia [1]. Shapiro syndrome (SS) was first described in 1969 as a clinical triad of episodic hypothermia, hyperhidrosis, and ACC [2]. A proposed reverse phenotype, known as RSS, involves episodic hyperthermia instead of hypothermia in association with ACC [1]. Although both conditions share a likely hypothalamic origin, RSS is far less common and underrecognized. Given the limited number of reported cases, clinical awareness remains low, leading to diagnostic delays and extensive workups. 

In this report, we present a case of RSS in a 33-year-old male patient, appearing with prolonged fever, and highlight the potential impact of levodopa in alleviating the patient’s symptoms. This case underscores the therapeutic role of dopaminergic agents in managing hypothalamic dysautonomia and expands our understanding of pharmacological interventions in RSS.

Τhis case report was previously presented as an oral presentation at the 36th Pan-Hellenic Congress of Neurology, on May 31, 2025.

## Case presentation

A 33-year-old male patient was admitted to the internal medicine clinic with a 10-day history of fever. The patient had a history of spastic tetraparesis since birth and was diagnosed with cerebral palsy at eight months of age. He was later diagnosed with epilepsy by the age of six, when magnetic resonance imaging (ΜRI) of the brain revealed partial agenesis of ACC. Over the past decade, he experienced multiple hospitalizations for fever, which lasted for several days to three weeks, often attributed to respiratory or urinary tract infections, despite no infectious agent being identified in the majority of the cases. This recurring pattern caused significant distress for the patient and his family. His chronic medications included levetiracetam, carbamazepine, lansoprazole, metoprolol, and baclofen, unchanged for the last five years. In the past 10 days, according to his mother, the patient experienced febrile episodes lasting 60 to 90 minutes, with daily axillary spiking fever of 38.6 °C. His parents mentioned no history of exposure to animals, insect bites, rash, arthralgia, abdominal discomfort, or weight loss. 

On admission, he was hemodynamically stable, with a weight and height of 55 kg and 165 cm, respectively, without an evident source of infection. Pulmonary examination was unremarkable, as was the examination of the ears, nose, and throat. Neurologic examination revealed spastic tetraparesis with increased tone in all four limbs, brisk tendon reflexes, bilateral Babinski signs, and sustained ankle clonus. Motor power was difficult to assess due to marked spasticity. He communicated with incomprehensible vocalizations, and he kept eye contact with his mother. Cardiac assessment revealed no murmurs, and the remainder of the physical examination was normal. Laboratory tests revealed low levels of inflammatory markers (CRP: 12 mg/L, normal <5 mg/L, negative procalcitonin), mild renal impairment, and hypoalbuminemia. The PCR test for SARS-CoV-2 came out negative. Abdominal ultrasound was found to be normal. Empirical antimicrobial therapy with the broad-spectrum antibiotics meropenem and vancomycin was initiated without remission of the febrile episodes. The febrile episodes occurred twice daily and reached up to 38.5 °C, lasted 60 to 90 minutes, sometimes accompanied by diaphoresis, without other autonomic abnormalities (blood pressure, heart rate). They were partially alleviated with paracetamol without achieving complete apyrexia, with a decrease to 37.5°C. At this time, the patient appeared exhausted with reduced communicative responsiveness, without other autonomic abnormalities; blood pressure and heart rate were within normal range.

**Figure 1:**
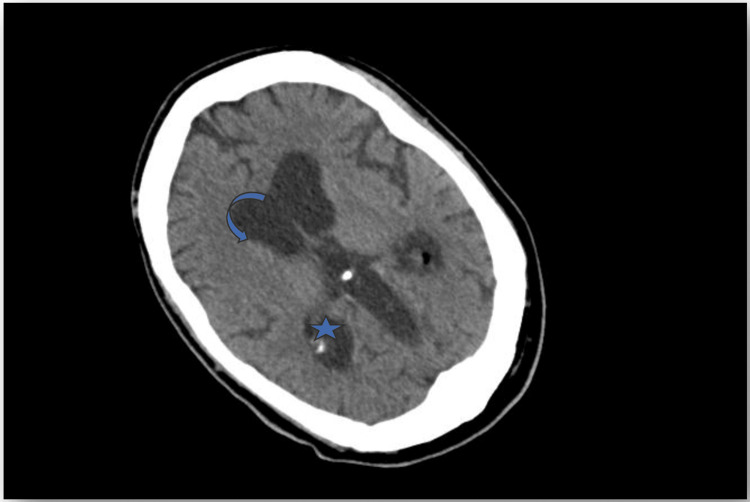
CT brain scan (axial view) of the patient, where features of partial ACC are noted; enlargement of the atria (asterisk), convex-shaped lateral portion of the anterior horns (open circle arrow). ACC: agenesis of the corpus callosum

**Figure 2:**
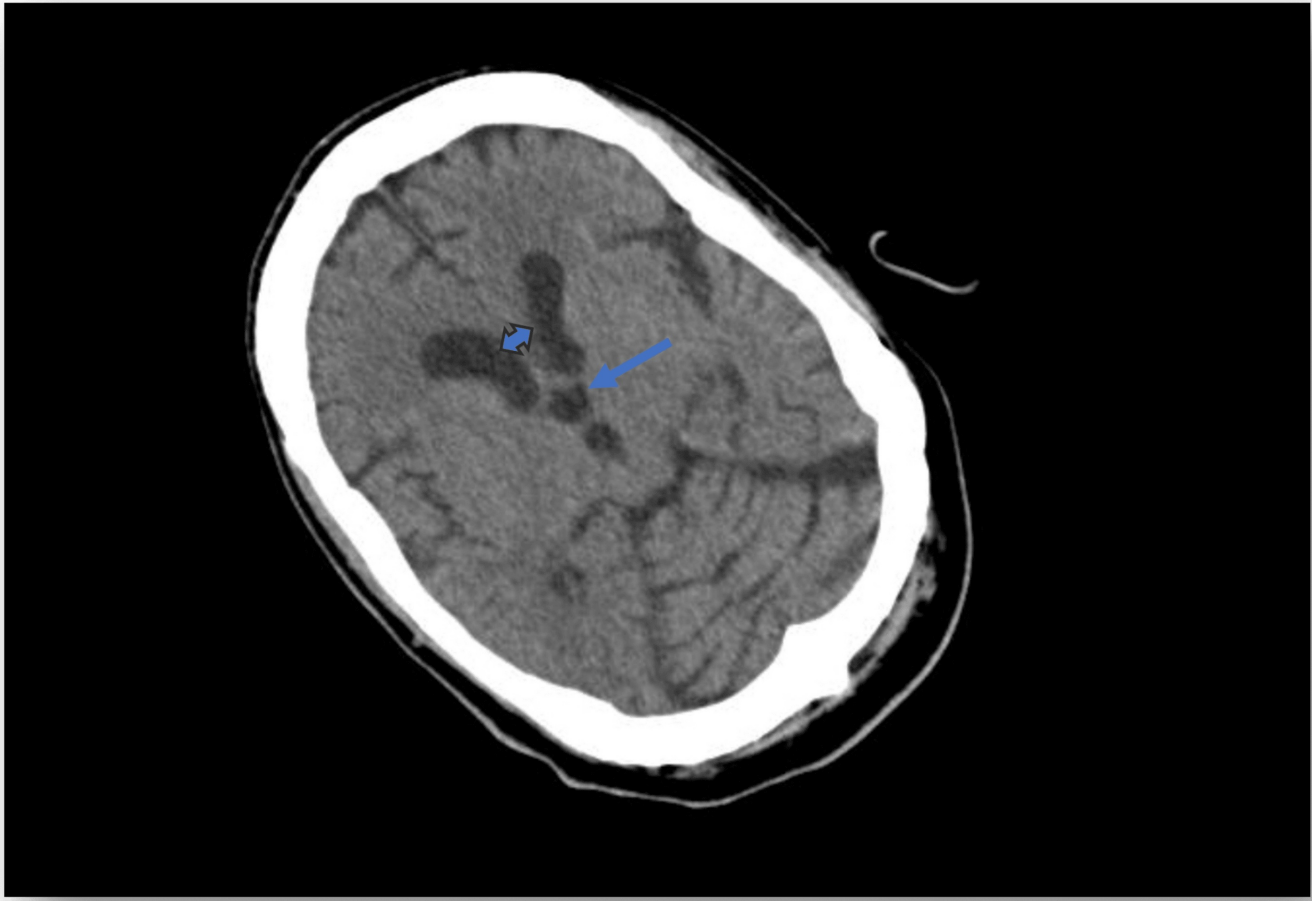
CT brain scan (axial view) of the patient. Note the dilatation of the third ventricle (dashed arrow) and the increased distance between the bodies of the lateral ventricles (double-headed arrow).

**Figure 3:**
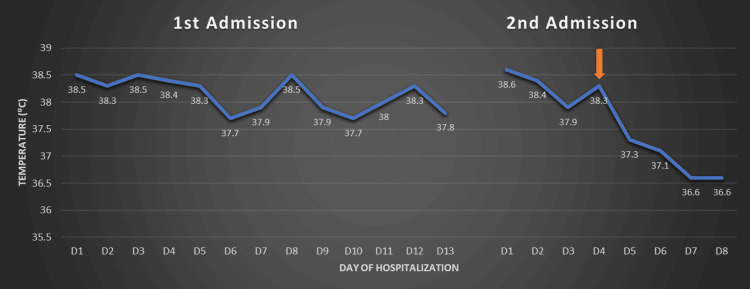
Temperature chart of the patient depicting the maximum temperature each day of the hospitalization, during the first and the second admission at the internal medicine clinic. The orange arrow indicates the initiation of levodopa; it is followed by a rapid decrease in temperature. The horizontal axis represents the days of hospitalization, while the vertical axis represents the temperature (°C). D1 : Day 1, D2: Day 2, etc.

Thyroid and immunological evaluations came out normal, while blood and urine cultures prior to antibiotic exposure revealed no pathogen. A thorough diagnostic workup for a fever of unknown origin was initiated. Serological tests for atypical pathogens and viral screenings were also negative. Peripheral blood smear and lumbar puncture did not reveal any pathological findings. CT scans of the chest and abdomen showed only a small right pleural effusion, not amenable to drainage, and right renal calculi, while echocardiography was unremarkable. CT scan of the brain revealed partial ACC, as noted in his personal medical history, with characteristic atrial enlargement, increased interventricular body spacing, dilatation of the third ventricle, and convex-shaped lateral part of the anterior horns (Figures 1, 2). Considering the possibility of a drug-induced fever, antibiotics were discontinued; however, the febrile episodes continued, with unchanged frequency and intensity. Given the history of partial ACC, RSS was suspected. Supportive measures, including cold showers and clothing, were applied, and after 12 days, the patient was discharged home; however, he was soon rehospitalized due to the same symptoms and family distress. In the absence of an identified infectious or other cause, treatment with levodopa was initiated at 50 mg, three times daily. This led to a rapid decline in febrile episodes until apyrexia was achieved and sustained, and the patient was discharged with noticeable clinical improvement, returning to his prior status (Figure 3). The initiation of levodopa therapy led to a clear and significant amelioration in the patient’s condition. During our follow-up in the following 12 months, the previously frequent and distressing episodes of unexplained hyperthermia disappeared, and the patient's family reported a substantial relief of discomfort, as well as longer intervals between hospitalizations.

## Discussion

Agenesis of the corpus callosum

The corpus callosum is the largest commissural fiber tract in the human brain, containing 200 million axons, and is responsible for the interhemispheric integration of sensory, motor, and cognitive information [3]. The development of the corpus callosum involves neuronal proliferation and migration, axonal outgrowth across the midline, and guidance by glial and molecular signals (e.g., Slit-Robo, Netrin-1/DCC pathways) [3,4]. It comprises four sections that develop from seven weeks’ gestation, beginning with the most anterior portion called the genu. Subsequently, and sequentially, the body and splenium develop with the rostrum (continuous with the genu extending inferiorly and then posteriorly) being the last section to develop. The corpus callosum has formed in its entirety by 20 weeks, after which gestation diagnosis can be achieved with accuracy. Insults during development that occur prior to 12 weeks result in complete agenesis, with partial agenesis (dysgenesis) occurring after this gestation [5]. Partial agenesis involves most commonly the body or the splenium [6]. 

ACC is one of the most common human brain malformations. It is a very heterogeneous group with a large variation in published prevalence based on a few population-based studies. It has an incidence of 0.5 to 70 in 10000, and its prevalence in children with developmental disabilities is about 230 in 10,000 (2.3%) [4]. It presents in one in 19,000 autopsies. A higher prevalence of 0.2-0.7% is also reported [7-9]. In subjects with impaired neurodevelopment, this defect is present in up to 1-3% of a given group [6,10]. ACC is more prevalent among males than females [3]. Diagnosis can be made prenatally in fetal ultrasound, during the second trimester, screening sonogram using the standard, three-axial-image assessment. The hallmarks of complete agenesis of the corpus callosum in ultrasound diagnosis are colpocephaly, i.e., focal dilatation of the posterior horns of the lateral ventricles, absent cavum septum pellucidum, and a high-riding third ventricle - filling the void left by the absent cavum septum pellucidum and corpus callosum. When abnormality of the corpus callosum is suspected, midline sagittal and coronal views of the corpus callosum through the fetal anterior fontanelle should be obtained to confirm the abnormality [11].

ACC can present as an isolated finding, although it is commonly linked to various syndromes, including Aicardi syndrome, Andermann syndrome, Shapiro syndrome, acrocallosal syndrome, and Menkes disease [12]. Additional related conditions include congenital anomalies from environmental causes like fetal alcohol syndrome, metabolic disorders, such as Krabbe disease, Dandy-Walker syndrome, and a variety of chromosomal abnormalities (e.g., 1p36 deletion, 8q24 duplication) [12].

Its clinical spectrum ranges from normal intelligence and minimal neurological findings to severe neurodevelopmental impairment, as was the case for our patient. Isolated ACC is often asymptomatic and may have mild cognitive or social deficits and subtle impairments in problem-solving, concept formation, and memory encoding. More recent findings point to interhemispheric transfer delays and deficits in bimanual coordination and visuospatial skills [13]. Syndromic/complex ACC usually involves developmental delay, intellectual disability, epilepsy (in 20-40% of cases), vision-related anomalies, motor coordination issues, feeding difficulties (in neonates), autism spectrum features, or behavioral dysregulation [7].

Shapiro syndrome

Shapiro syndrome was first described in 1969 by William R. Shapiro et al. in two women (46- and 34-year-old) with recurrent episodes of spontaneous hypothermia (as low as 30°C), accompanied by hyperhidrosis (excessive sweating), somnolence, and bradycardia [2]. Brain imaging (pneumoencephalography at the time) revealed anomalies in both patients associated with agenesis of the corpus callosum. Shapiro et al. proposed the term “spontaneous periodic hypothermia with corpus callosum agenesis” and hypothesized diencephalic epilepsy as a potential mechanism, although the electroencephalogram did not show epileptiform discharges. These cases established the syndrome as a rare disorder involving hypothalamic thermoregulation, later termed “Shapiro syndrome.” Core symptoms of the syndrome include spontaneous hypothermia (often 32-35°C), profuse sweating, lethargy or altered mental status, partial or complete ACC (confirmed by MRI), as well as other associated features such as bradycardia or other autonomic dysfunction, seizures (in some cases), developmental delay (especially in pediatric patients) and behavioral disturbances. Episodes may last from hours to days and recur intermittently, with variable triggers (e.g., stress, infections).

Approximately 50 cases of SS have been reported in the literature [14-16]. A recent review by Linan Ren et al. suggested that spontaneous periodic hypothermia is a hallmark of typical and variant SS and that hyperhidrosis and ACC are supportive features not observed in all cases [16]. Moreover, the researchers found that the clinical manifestations of the syndrome included autonomic nervous dysfunction, complications related to hypothermia, and complications related to ACC. 

Reverse Shapiro syndrome

To our knowledge, six RSS cases have been reported in the literature so far [1,17-21].

The first documented case of RSS was published in 1994 by Hirayama K et al. [1]. The report describes a 14-year-old girl with ACC and recurrent episodes of hyperthermia, without other neurological or physical abnormalities. Neuroimaging did not reveal any structural abnormalities of the diencephalon, while endocrine evaluations and electroencephalograms were regular. Hyperthermia resolved with a low dose of levodopa (200 mg/day), but a higher dose (400 mg/day) induced hypothermia, compared to our case, where 150 mg/day of levodopa achieved complete apyrexia. The same dose was administered to our patient, leading to apyrexia as well. Until our report, this was the only other case of RSS not pertaining to an infant.

In 2005, Lin and Wang reported a case involving a nine-month-old female infant with RSS, where treatment with levodopa (at 7.5 mg/kg/day) with carbidopa, and cyproheptadine hydrochloride (serotonin antagonist) failed to alleviate the symptoms [17]. Subsequently, in 2012, Guha et al. described the clinical features of RSS in a three-month-old girl who was being evaluated for fever of unknown origin [18]. Despite the initial administration of a low dose of levodopa, the hyperthermia persisted until the patient reached 14 months of age, during which period of time, only physical cooling measures, such as tepid sponging, were employed.

Topçu et al., in 2013, identified ACC in a 3.5-year-old female presenting with recurrent febrile episodes and vomiting, alternating with episodes of hypothermia and diaphoresis [19]. After excluding other potential causes of fever, the patient was diagnosed with RSS. Cyproheptadine hydrochloride therapy was found to be ineffective. All the reported cases so far had dealt with SS and RSS as two different entities, yet, innovatively enough, Topçu et al. suggested that these two diseases represent different aspects of the same underlying disorder since the same patient can have episodes of hypo- and hyperthermia. This is further supported by the similar therapeutic response observed in both conditions.

Mansour et al. later reported a case involving a six-month-old male who had been prenatally diagnosed with ACC and exhibited recurrent fevers of indeterminate cause [20]. In contrast to prior reports, this patient responded positively to cyproheptadine hydrochloride therapy of 0.25 mg/kg/day, thrice daily, which successfully controlled the hyperthermic episodes, with a follow-up of six months.

The sixth case was reported by Dag et al. in 2019, in India, and involved a six-month-old girl presenting with fever of unknown origin and neurodevelopmental delay [21]. MRI findings confirmed diffuse hypoplasia of the corpus callosum and delayed myelination. It is the only published case so far, along with ours, with partial agenesis of the corpus callosum. Despite treatment with cyproheptadine and methylprednisolone, the patient showed no clinical improvement, highlighting the limited efficacy of current therapies.

Table 1 summarizes each patient's key demographic characteristics, principal imaging or clinical findings, the treatments administered, and the authors' conclusions regarding each case. 

**Table 1:** Summary of published RSS cases. ACC: agenesis of corpus callosum, EEG: electroencephalogram, WEST epilepsy: defined by the triad of epileptic spasm, an interictal EEG pattern termed hypsarrhythmia, and developmental stagnation or regression, RSS: reverse Shapiro syndrome

Authors (year)	Patient info	Symptoms	Findings	Treatment	Conclusion
Hiraya-ma et al. (1994) [1]	14-year-old female	Recurrent hyperthermia	Complete ACC; normal EEG	Levodopa effective at low doses; high doses caused hypothermia	Dopaminergic denervation of hypothalamic thermoregulation
Lin and Wang (2005) [17]	9-month-old female	Fever of unknown origin	Complete ACC; normal EEG	Levodopa with carbidopa and cyproheptadine ineffective	Possible dopaminergic denervation of the hypothalamus
Guha et al. (2012) [18]	3-month-old female	High-grade fever, one convulsion	Complete ACC	Levodopa ineffective	Thermo-dysregulation due to ACC
Topcu et al. (2013) [19]	3.5-year- old female	Recurrent fever, vomiting, hypothermia, diaphoresis	Complete ACC	Cyproheptadine ineffective	ACC-related hypothalamic dysfunction, both SS and RSS may reflect the same disorder
Mansour et al. (2017) [20]	6-month-old male	Recurrent hyperthermia	Complete ACC; WEST epilepsy, neuro-developmental delay	Cyproheptadine effective	Epileptic origin of hypothalamic dysregulation
Dag et al (2019) [21]	6-month-old female	Fever of unknown origin, two convulsions	Advanced hypoplasia of the corpus callosum, delayed myelination, neuro-developmental delay	Cyproheptadine, methyl-prednisolone ineffective	RSS can be accompanied by hypoplasia of the corpus callosum.

To our knowledge, the current case represents the seventh documented instance in the literature, presenting as a fever of unknown origin. Distinguishing features of our case were that our patient was substantially older (33 years old) and the fact that he responded to levodopa administration, with a successful follow-up of one year. The only other case of RSS that responded to levodopa was the one described by Hirayama K et al. [1], suggesting that the underlying pathophysiological mechanisms of the syndrome may differ between infants and surviving adolescents or adults with RSS. Consequently, the option of reinitiation of levodopa treatment in later stages of a patient's life should be explored. 

In the aforementioned cases, a magnetic resonance imaging of the brain (MRI) was conducted, which showed colpocephaly, a high-positioned roof of the third ventricle, and enlargement of the posterior horns. These features are characteristic of agenesis of the corpus callosum [22,23]. Only in the case of Dag et al. MRI revealed diffuse hypoplasia of the corpus callosum in the T2-weighted image and hypotense genu of the corpus callosum in the T1-weighted image. We acknowledge that MRI is the modality of choice for evaluating the imaging characteristics of corpus callosum agenesis, due to its multiplanar capability and superior soft-tissue contrast. However, despite our best efforts, the patient was unable to undergo an MRI examination because of an inability to remain still for the duration required. In this context, a CT scan was performed, which can also support the diagnosis of corpus callosum agenesis. On the axial images, the lateral ventricles appear parallel and do not converge anteriorly. Coronal images demonstrate a high-riding third ventricle and indentation along the medial borders of the frontal horns, producing the characteristic “Viking horns” or “bull’s horns” appearance. In addition, although less prominent in the case of our patient, both axial and coronal views reveal dilated occipital horns, consistent with colpocephaly.

Based on the aforementioned cases, one may reasonably conclude that RSS may present as a cause of fever of unknown origin in patients with congenital central nervous system anomalies, significantly impact the patient's quality of life, as well as contribute to caregiver burden, underscoring the importance of timely detection and clinical management. Prompt identification and targeted treatment of RSS with levodopa not only led to a clear improvement in our patient's recurrent hyperthermia but also had a broader impact on his quality of life and the well-being of his family. Prior to the diagnosis, the patient experienced frequent discomfort and functional impairment, while his caregivers faced uncertainty and anxiety, leading to consecutive hospitalizations in search of help and an explanation. Establishing the diagnosis helped avoid future unnecessary investigations, admissions, and interventions, thereby reducing healthcare burden and emotional stress.

Potential pathophysiological mechanisms behind SS and RSS

Hypothalamic Dysregulation

Thermoregulation is governed by a complex neural network involving the hypothalamus, limbic system, brainstem, spinal cord, and sympathetic ganglia, with the hypothalamus serving as the central control center [24]. The hypothalamus regulates core body temperature via the autonomic nervous system. Disruption in central thermoregulatory pathways, possibly due to aberrant interhemispheric communication or intrinsic hypothalamic dysfunction, may result in paroxysmal sympathetic hyperactivity or dysregulation, hypothermia with paradoxical hyperhidrosis, and altered sensorium during attacks [25]. Within the hypothalamus, two distinct thermoregulatory centers exert opposing effects, potentially resulting in either hypothermia or hyperthermia depending on the balance of their activity. Damage to the anterior hypothalamic center, which regulates heat dissipation, results in hyperthermia, while damage to the posterior hypothalamic center, responsible for heat conservation, leads to hypothermia [25]. One proposed mechanism underlying these thermal disturbances involves structural abnormalities in dopaminergic pathways responsible for temperature control, along with heightened sensitivity of dopamine receptors [25,26]. This dysfunction may ultimately contribute to dopaminergic denervation. Furthermore, neuronal degeneration in affected individuals may be accompanied by fibrillary gliosis within the hypothalamus. Hyperhidrosis, on the other hand, is characterized by excessive sweating and generally results from heightened sympathetic nervous system activity, which may be precipitated by various conditions, including central nervous system disorders, infections, tumors, endocrine abnormalities, and certain medications.

In SS and RSS, it is hypothesized that there is a disrupted interhemispheric communication, although episodes of hypothermia or hyperthermia, respectively, occur without any visible structural damage to brain regions involved in regulating body temperature [27,28]. It is unlikely that the symptoms are solely due to agenesis of the corpus callosum, as most individuals with this brain anomaly do not experience these symptoms [29]. Moreover, surgical callostomy does not typically lead to the syndrome, and some familial cases show a Shapiro-like presentation despite having normal brain imaging [27,28]. Alternatively, it is possible that the hypothalamic dysregulation may arise secondary to underlying epileptic syndromes [16,20,30].

Neurotransmitter Imbalances

Autonomic disturbances in SS suggest a role for neurotransmitter imbalance [16,28,31,32]. Dysregulation of serotonin and dopamine levels has been implicated, particularly in shivering thresholds and thermoregulatory pathways within the hypothalamus. These findings, along with the diverse mechanisms of medications that seem to alleviate symptoms, such as sympatholytics, serotonin blockers, dopamine agonists, anticholinergics, and anticonvulsants, indicate that the disorder may stem from molecular-level dysfunction within a complex neural network involving multiple neurotransmitter systems [1,32]. Positive clinical responses to serotonergic agents (e.g., cyproheptadine) in some cases further support this hypothesis [20].

Melatonergic Involvement

Melatonin, chiefly secreted by the pineal gland, influences circadian rhythms and thermoregulation. In one SS case, elevated nocturnal melatonin levels correlated with symptom exacerbation, implicating hypermelatonemia in SS pathogenesis [16,30,33]. Nevertheless, this remains anecdotal, and further data are required to justify this possible mechanism. Notably, melatonin secretion is modulated by the suprachiasmatic nucleus, supporting its link to hypothalamic dysfunction.

**Figure 4:**
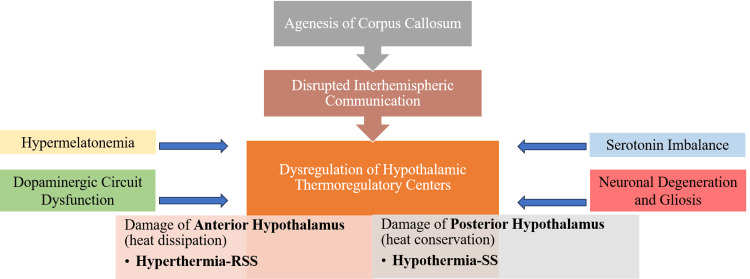
Proposed pathophysiological mechanisms of SS and RSS. Agenesis of the corpus callosum is believed to impair interhemispheric communication, potentially resulting in dysregulation of hypothalamic thermoregulatory mechanisms. Specifically, damage to the anterior hypothalamus has been associated with hyperthermia, which may contribute to the development of RSS. Conversely, injury to the posterior hypothalamus—responsible for heat retention—can lead to hypothermia and subsequent SS. In parallel, additional factors are thought to exacerbate this thermoregulatory imbalance, including elevated melatonin levels (hypermelatonemia), dysfunction in dopaminergic pathways, serotonin imbalance, as well as progressive neuronal degeneration and gliosis. This figure constitutes original work produced by the authors and has not been adapted from external sources. RSS: reverse Shapiro syndrome, SS: Shapiro syndrome

In sum, SS and RSS likely arise from complex, multifactorial dysfunction centered on hypothalamic regulation, with potential contributions from melatonin and neurotransmitter systems. The exact pathophysiologic mechanisms have so far been only hypothetical (Figure 4). Despite the growing evidence, a definitive diagnostic biomarker remains elusive.

Treatment

Although no definitive treatment currently exists for either SS or RSS, several therapeutic approaches have been attempted to manage episodes of hypothermia and hyperthermia. For hypothermic cases, interventions have included dopamine agonists (such as levodopa), dopamine antagonists (e.g., chlorpromazine hydrochloride), α2-adrenergic agonists (e.g., clonidine), and sympathectomy [17-21]. Hirayama et al. reported that administering 200 mg/day of levodopa successfully reduced fever, but increasing the dose to 400 mg/day induced hypothermia [1]. Conversely, in the case reported by Ling and Wang, neither levodopa (a dopamine agonist) nor cyproheptadine hydrochloride (a serotonin antagonist) effectively managed hyperthermia [17]. Similarly, both Guha et al. and Topçu et al. observed no improvement in hyperthermia with cyproheptadine hydrochloride treatment [18,19]. However, Mansour et al. successfully controlled hyperthermia by administering cyproheptadine hydrochloride at a dose of 0.25 mg/kg/day, administered every eight hours [20]. In the last reported case, the patient initially received intravenous methylprednisolone (2 mg/kg/day) for five days, but due to a lack of improvement, the therapy was discontinued [21]. The patient was then switched to oral cyproheptadine hydrochloride (0.25 mg/kg/day, q8h), but hypothermia persisted after 72 hours, leading to cessation of that treatment as well. In our case, however, levodopa led to complete remission of symptoms, one year later, and continuing. This positive response to dopaminergic therapy supports the hypothesis of hypothalamic dopaminergic dysfunction in the pathophysiology of reverse Shapiro syndrome. Moreover, it reinforces the importance of early recognition and therapeutic trial of levodopa in suspected cases. The resolution of symptoms with such a targeted treatment underlines the diagnostic and therapeutic value of this rare syndrome.

## Conclusions

RSS remains a rare and underrecognized clinical entity. While very few cases have been reported in the literature, it is essential to emphasize that the condition likely remains underdiagnosed rather than exceptionally rare. ACC, which is the hallmark feature of both SS and RSS, is not an exceedingly rare anomaly; it is one of the most common brain malformations. It is plausible that a subset of patients with ACC may develop thermoregulatory disturbances that go unnoticed or are misattributed to other causes. They may present recurrent episodes of unexplained fever, often leading to extensive diagnostic work-ups and unnecessary hospitalizations. Such cases are frequently classified as "fever of unknown origin," with clinicians overlooking hypothalamic dysregulation as a potential source. Our case illustrated that treatment with levodopa produced a notable therapeutic response, with significant resolution of symptoms, most likely attributable to the restoration of dopaminergic balance within the hypothalamus. Early recognition not only alleviates the patient’s symptoms and distress but also spares families and caregivers from ongoing uncertainty, while reducing the burden on healthcare systems by avoiding redundant investigations and admissions.
